# Acidic Amino Acids in the First Intracellular Loop Contribute to Voltage- and Calcium- Dependent Gating of Anoctamin1/TMEM16A

**DOI:** 10.1371/journal.pone.0099376

**Published:** 2014-06-05

**Authors:** Qinghuan Xiao, Yuanyuan Cui

**Affiliations:** 1 From the Department of Ion Channel Pharmacology, School of Pharmacy, China Medical University, Shenyang, China; 2 Department of Cell biology, School of Medicine, Emory University, Atlanta, Georgia, United States of America; Monell Chemical Senses Center, United States of America

## Abstract

Anoctamin1 (Ano1, or TMEM16A) is a Ca^2+^-activated chloride channel that is gated by both voltage and Ca^2+^. We have previously identified that the first intracellular loop that contains a high density of acidic residues mediates voltage- and calcium-dependent gating of Ano1. Mutation of the four consecutive glutamates (_444_EEEE_447_) inhibits the voltage-dependent activation of Ano1, whereas deletion of these residues decreases apparent Ca^2+^ sensitivity. In the present study, we further found that deletion of _444_EEEEEAVKD_452_ produced a more than 40-fold decrease in the apparent Ca^2+^ sensitivity with altered activation kinetics. We then systematically mutated each acidic residue into alanine, and analyzed the voltage- and calcium dependent activation of each mutation. Activation kinetics of wild type Ano1 consisted of a fast component (τ_fast_) that represented voltage-dependent mode, and a slow component (τ_slow_) that reflected the Ca^2+^-dependent modal gating. E444A, E445A, E446A, E447A, E448A, and E457A mutations showed a decrease in the τ_fast_, significantly inhibited voltage-dependent activation of Ano1 in the absence of Ca^2+^, and greatly shifted the G-V curve to the right, suggesting that these glutamates are involved in voltage-gating of Ano1. Furthermore, D452A, E464A, E470A, and E475A mutations that did not alter voltage-dependent activation of the channel, significantly decreased Ca^2+^ dependence of G-V curve, exhibited an increase in the τ_slow_, and produced a 2–3 fold decrease in the apparent Ca^2+^ sensitivity, suggesting that these acidic residues are involved in Ca^2+^-dependent gating of the channel. Our data show that acidic residues in the first intracellular loop are the important structural determinant that couples the voltage and calcium dependent gating of Ano1.

## Introduction

−Calcium-activated chloride channels (CaCCs) play diverse and important roles in cellular functions, including epithelial secretion, regulation of smooth muscle contraction, control of neuronal and cardiac excitability, sensory transduction, and nociception [1]–[3]. In 2008, Anotamin1 (Ano1 or TMEM16A) and Anoctamin2 (Ano2, or TMEM16B), two of the 10 members in the Ano family, were identified as CaCCs by three independent laboratories [4]–[6]. Since then, Ano1 and Ano2 have been found to be essential components of native CaCCs in many cells, including epithelial cells in the salivary gland, pancreatic gland, airway, gastrointestinal tract [4], [7]–[9], airway and vascular smooth muscle cells [8], [10]–[12], sensory neurons in the dorsal root ganglia [13], olfactory neurons [14], and interstitial cells of Cajal [15], [16]. However, it remains controversial to claim that other Ano family members are anion channels [1], [3]. For example, Suzuki et al. have reported that Ano6 (TMEM16F) functioned as a Ca^2+^-dependent phospholipid scramblase, but not as a chloride channel [17], [18]. Other researchers have found that Ano6 forms a Ca^2+^-activated anion channel [19], [20]. In addition, it has been reported that Ano6 constitutes a Ca^2+^-activated nonselective cation channel [21]. Despite these controversies, it appears that Ano family mediates Ca^2+^-activated biological functions.

Although recent studies have investigated the mechanisms underlying Ca^2+^ regulates Ano1, the mechanisms remains unclear. It has been reported that calmodulin (CaM) regulates Ano1 via at least two binding motifs in the channel [22], [23]. In excised patches where Ca^2+^/CaM mediated increase in HCO_3_^−^ permeability of Ano1 is lost [23], Ano1 can be directly activated by Ca^2+^ [23], [24], suggesting that a Ca^2+^-binding site on the channel may be responsible for activation of Ano1 by Ca^2+^. The explicit Ca^2+^-binding site in Ano1 has not been identified. We have previously found that the first intracellular loop that contains five consecutive acidic residues are critical for both calcium- and voltage-dependent gating of the channel [24]. A splice variant of Ano1 lacks four amino acids (_448_EAVK_451_) in the first intracellular loop decreases the apparent Ca^2+^ sensitivity [24], [25]. Furthermore, two acidic amino acids E702 and E705, which are identified to be intracellular in a revised topology model, contribute to Ca^2+^ gating of the channel [26]. It appears that Ano1 may have multiple calcium binding sites or a binding site involving in disparate regions on the channel.

Acidic amino acids are known to contribute to coordinate Ca^2+^ in several Ca^2+^-binding proteins [27], [28]. The first intracellular loop of Ano1 contained a high density of acidic amino acids, including five consecutive acidic residues [24]. We have previously identified that deleting _448_EAVK_451_ in the first intracellular loop dramatically decreases apparent Ca^2+^ sensitivity of Ano1, and mutating the adjacent _444_EEEE_447_ alters voltage-dependent activation of Ano1 without significant changes in the apparent Ca^2+^ sensitivity [24]. These findings suggest that the acidic residues in the first intracellular loop may be involved in voltage- and Ca^2+^-dependent gating of Ano1. Here, we mutated each acidic residue to alanine in an attempt to identify the role of each acidic residue in voltage- and calcium-dependent gating of Ano1. We found that the five consecutive glutamates _444_EEEEE_448_ and E457 were critical for voltage-dependent gating of Ano1, and acidic residues D452, E464, E470, and E475 following _448_EAVK_451_ contribute to regulation of Ano1 by Ca^2+^.

## Materials and Methods

### Construct and Molecular Biology

Ano1(*ac*) tagged with enhanced green fluorescent protein (EGFP) was obtained from Dr. U. Oh (Seoul National University, Korea). Site-specific mutations were generated using PCR-based mutagenesis (Quickchanger, Agilent Technologies). All constructs were confirmed by sequencing.

### Cell Culture and Transfection

HEK-293 cells (American Type Culture Collection, Manassas, VA, USA) were cultured in DMEM supplemented with 10% fetal bovine serum and 0.5% penicillin-streptomycin at 37°C. Low-passage HEK cells were transiently transfected with Fugene-6 (Roche) with 1 µg Ano1 per 35-mm dish. Cells were also transfected with 1 µg of pEGFP for fluorescence detection. Transfected cells were plated at low density and investigated between 24 and 72 h after transfection.

### Electrophysiology

Transfected cells were identified by EGFP fluoresce. The electrophysiological recordings were performed in whole-cell and inside-out patch clamp configurations. Patch pipettes had resistances of 2–4 MΩ. Data were acquired by an Axopatch 200B amplifier controlled by Clampex 9 via a Digidata 1322 A data acquisition system (Molecular Device, Sunnyvale, CA, USA). For whole-cell recordings, the “0” Ca^2+^ pipette solution contained (in mM): 146 CsCl, 2 MgCl_2_, 5 EGTA, 10 sucrose, and 8 HEPES, pH 7.3 adjusted with NMDG. The “high” Ca^2+^ pipette solution contained 5 mM Ca^2+^-EGTA instead of EGTA, which supplied free Ca^2+^ of about 25 µM. Solutions contained different concentrations of free Ca^2+^ were made by mixing the “0’ Ca^2+^ and “high” Ca^2+^ solutions. Free Ca^2+^ concentrations were verified using Fure-2 and Fura 6F (Invitrogen). The standard external solution contained (in mM): 140 NaCl, 4 KCl, 2 CaCl_2_, 1 MgCl_2_, 10 glucose, and 10 HEPES (pH 7.3). For voltage-dependent activation, cells were voltage clamped with 50-ms voltage steps from −100 to +200 mV in 20-mV increments. For calcium-dependent activation, cells were voltage clamped with 750-ms voltage steps from −100 to +100 mV. For excised patches, the standard external solution was used as pipette solution. For better buffering the Ca^2+^, 3.5 mM Dibromo-BAPTA (5,5′-dibromo-1,2-bis(2-aminophenoxy)ethane-N,N,N′,N′-tetraacetic acid, K_d_ = 1.6 µM, Molecular Probe, Inc) was used in the excised patch to make 1 µM and 2 µM free Ca^2+^ concentrations. Patches were voltage clamped with 300-ms voltage steps from −160 to +200 mV in 20-mV increments at 10-s intervals. Osmolarity was adjusted with sucrose to 305 mOsm for all solutions.

### Data Analysis

We used Origin7 software for the calculations and graphical presentations. The activation kinetics of currents traces were fitted by two exponentials with Clampfit9, and visually inspected for proper fit. Dose-response curve of wild type Ano1 and mutations were generated by plotting the steady state current densities at +100 versus Ca^2+^ concentrations. The values of EC_50_ and Hill coefficients were calculated by fitting the current densities with the Hill equation, I/Imax = 1/(1+ (EC_50_/[Ca^2+^])^n^). G-V curves were generated from amplitudes of tail currents measured 200 µs after repolarization to −100 mV. G-V relations were fitted with a Boltzmann function, G/G_max_ = 1/1+exp(- (V-V_0.5_) zF/RT), where z is the equivalent gating charge, V_0.5_ is the voltage at half-maximal activation, F is Faraday’s constant, R is the gas constant, and T is temperature. Z values were calculated from the slope of the G-V curve. Results were represented as mean±SEM. One-way analysis of variance (ANOVA) were performed to compare the differences between the mutations and wild type Ano1, followed by post-hoc Bonferroni test. A value of *p*<0.05 was considered statistically significant.

## Results

**Figure 1:**
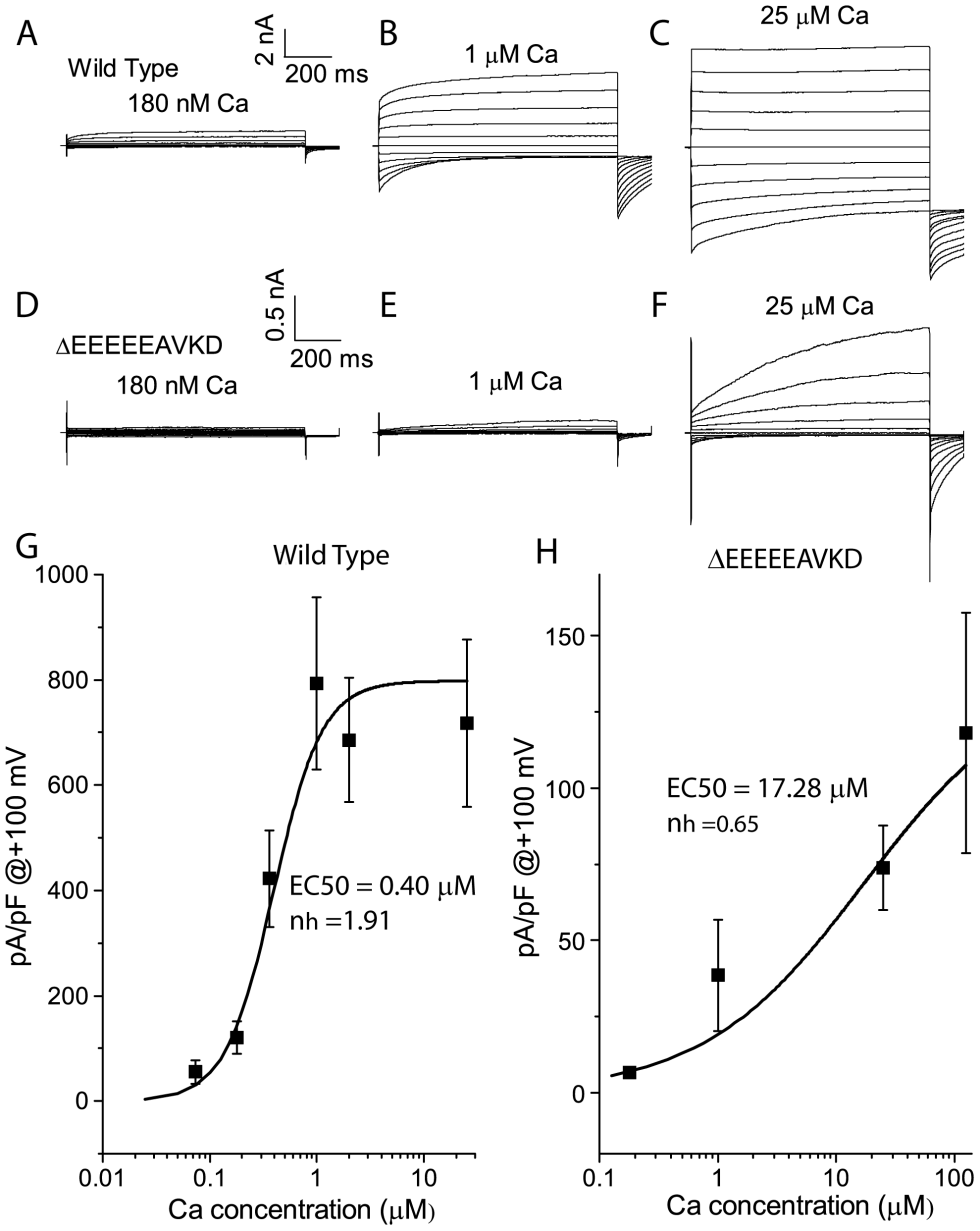
Activation of wild type and Δ_444_EEEEEAVKD_452_ Ano1 by Ca^2+^ in the whole-cell recordings. A–F. Representative traces of wild type (A–C) and Δ_444_EEEEEAVKD_452_ (D–F) Ano1 activated by Ca^2+^ concentrations ranging from 180 nM to 25 µM. Cells were voltage clamped from a holding potential of 0 mV to various potentials between −100 mV to +100 mV in 20 mV increments for 700 ms, followed by a 100-ms step to −100 mV. G.H. The steady state current densities at +100 were plotted versus Ca^2+^ concentrations from wild type (G) and Δ_444_EEEEEAVKD_452_ (H). The plots were fitted to Hill equations. n = 4–9 cells.

The first intracellular loop of Ano1 contains a high concentration of acidic amino acids, including five consecutive glutamates (_444_EEEEE_448_). We previously found that substituting the first four glutamates with alanines (_444_EEEE/AAAA_447_) inhibited the voltage-dependent activation of Ano1 without changes in the apparent Ca^2+^ sensitivity, whereas deleting _448_EAVK_451_ (ΔEAVK mutation) decreased the apparent Ca^2+^ sensitivity with enhanced voltage-dependent activation of Ano1 [24]. The increase in the voltage-dependent activation of the ΔEAVK mutation was completely abolished by deleting _444_EEEEEAVKD_452_ (Δ_444_EEEEEAVKD_452_) [24], suggesting that these consecutive glutamates in this region were critical for voltage-dependent gating of Ano1. We further tested the Ca^2+^ sensitivity of the Δ_444_EEEEEAVKD_452_ mutation, compared with wild type (WT) (Fig. 1). At intracellular Ca^2+^ concentration ([Ca^2+^]_i_) <1 µM, WT Ano1 was strongly outwardly rectifying. At 25 µM Ca^2+^, outward rectification was greatly reduced, and the currents were much less time-dependent (Fig. 1A–C) [24]. For Δ_444_EEEEEAVKD_452_ mutation, very little current was activated at [Ca^2+^]_i_ ≤1 µM (Fig. 1 D,E). At 25 µM Ca^2+^, the current densities were only ∼20% as large as WT (Fig. 1F). In contrast to WT, the current of the mutation exhibited pronounced time-dependent activation and deactivation and outward rectification (Fig. 1F). The Δ_444_EEEEEAVKD_452_ mutation exhibited the apparent EC_50_ for Ca^2+^ of 17.28 µM, which was approximately 40-fold higher than that of the WT (0.4 µM) (Fig. 1G,H). These findings further demonstrated that the acidic acids in the first intracellular loop were critical for voltage- and calcium-dependent gating of Ano1.

**Figure 2:**
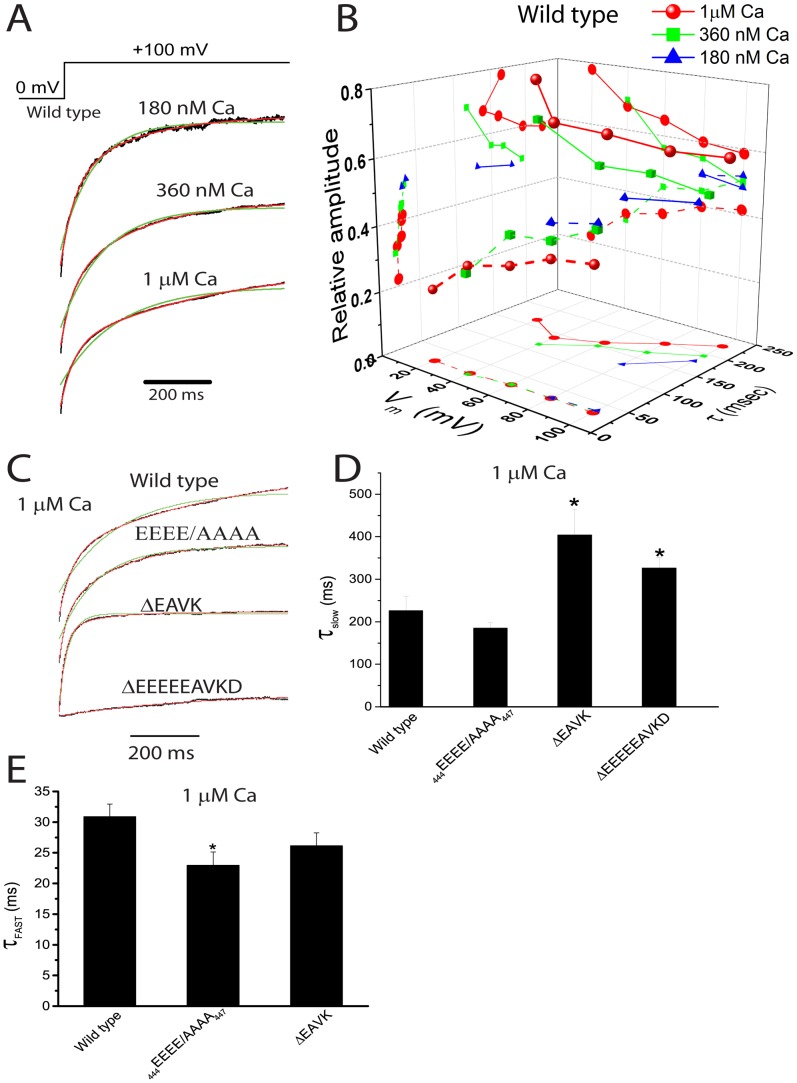
Activation kinetics of Ano1 by Ca^2+^ and voltage. A. Representative currents of wild type Ano1, activated by 180 µM Ca^2+^ at +100 mV from a holding potential of 0 mV (voltage protocol shown above). The currents were fitted to single exponentials (superimposed in green lines) and to two exponentials (superimposed in red lines). B. The relationship of the fast (τ_fast_) (dash lines) and slow (τ_slow_) (solid lines) components of the activation time constant of wild type Ano1 activated by 180 nM (blue triangle), 360 nM (green square), and 1 µM (red circle) Ca^2+^ at voltages between +20 mV to +100 mV from a holding potential of 0 mV (n = 4–6 cells). C. Representative currents of wild type, _444_EEEE/AAAA_447_, ΔEAVK, and Δ_444_EEEEEAVKD_452_ activated by 1 µM Ca^2+^ at +100 mV from a holding potential of 0 mV. The currents were fitted to single exponentials (superimpose in green lines) and to two exponentials (superimposed in red lines). D.E. The time constant of the slow (D) and fast (E) components of wild type, _444_EEEE/AAAA_447_, ΔEAVK, and Δ_444_EEEEEAVKD_452_ activated by 1 µM Ca^2+^ at +100 mV from a holding potential of 0 mV. n = 4–8 cells; *p<0.05 vs wild type.

We further characterized the kinetics of current activation at different Ca^2+^ concentrations for WT, _444_EEEE/AAAA_447,_ ΔEAVK, and Δ_444_EEEEEAVKD_452_ Ano1. For WT, the activation of Ano1 current was usually poorly fit to a single exponential, but was well-fit by two exponentials, suggesting that the activation of Ano1 in the presence of Ca^2+^ requires at least two conformational changes (Fig. 2A). Activation kinetics exhibited a shallow dependence on both Ca^2+^ and on voltage (Fig. 2B). The τ_fast_ slowed with depolarization and accelerated with increasing [Ca^2+^], whereas the τ_slow_ responded in the opposite way. The relative amplitude of the fast component increased with depolarization and the relative amplitude of the slow component increased with increasing [Ca^2+^] (Fig. 2B), suggesting that the fast component represented voltage-dependent mode, whereas the slow component reflected the Ca^2+^-dependent modal gating.

The activation of Δ_444_EEEEEAVKD_452_ current was well-fit to a single exponential (Fig. 2C). The Δ_444_EEEEEAVKD_452_ mutation, which showed a decrease in the apparent Ca^2+^ sensitivity (Fig. 1H), had a significantly slower τ_slow_ compared with WT (Fig. 2D), suggesting that decreased Ca^2+^ sensitivity was associated with a slow τ_slow_ of the channel. In agreement with this finding, compared with WT, the ΔEAVK mutation, which exhibited decreased Ca^2+^ sensitivity [24], also exhibited a significantly slower τ_slow_, whereas the _444_EEEE/AAAA_447_ mutation, which did not alter the apparent Ca^2+^ sensitivity [24], had a similar τ_slow_ (Fig. 2D). In addition, the fast component was completely abolished by the Δ_444_EEEEEAVKD_452_ mutation (Fig. 2C), which exhibited no voltage-dependent activation [24]. The _444_EEEE/AAAA_447_ mutation, which showed decreased voltage-dependent activation of the channel [24], exhibited a significantly faster τ_fast_ compared with wild type (Fig. 2E). These results suggested that altered voltage-dependent activation of the channel was associated with a change in the τ_fast_ of the channel.

**Figure 3:**
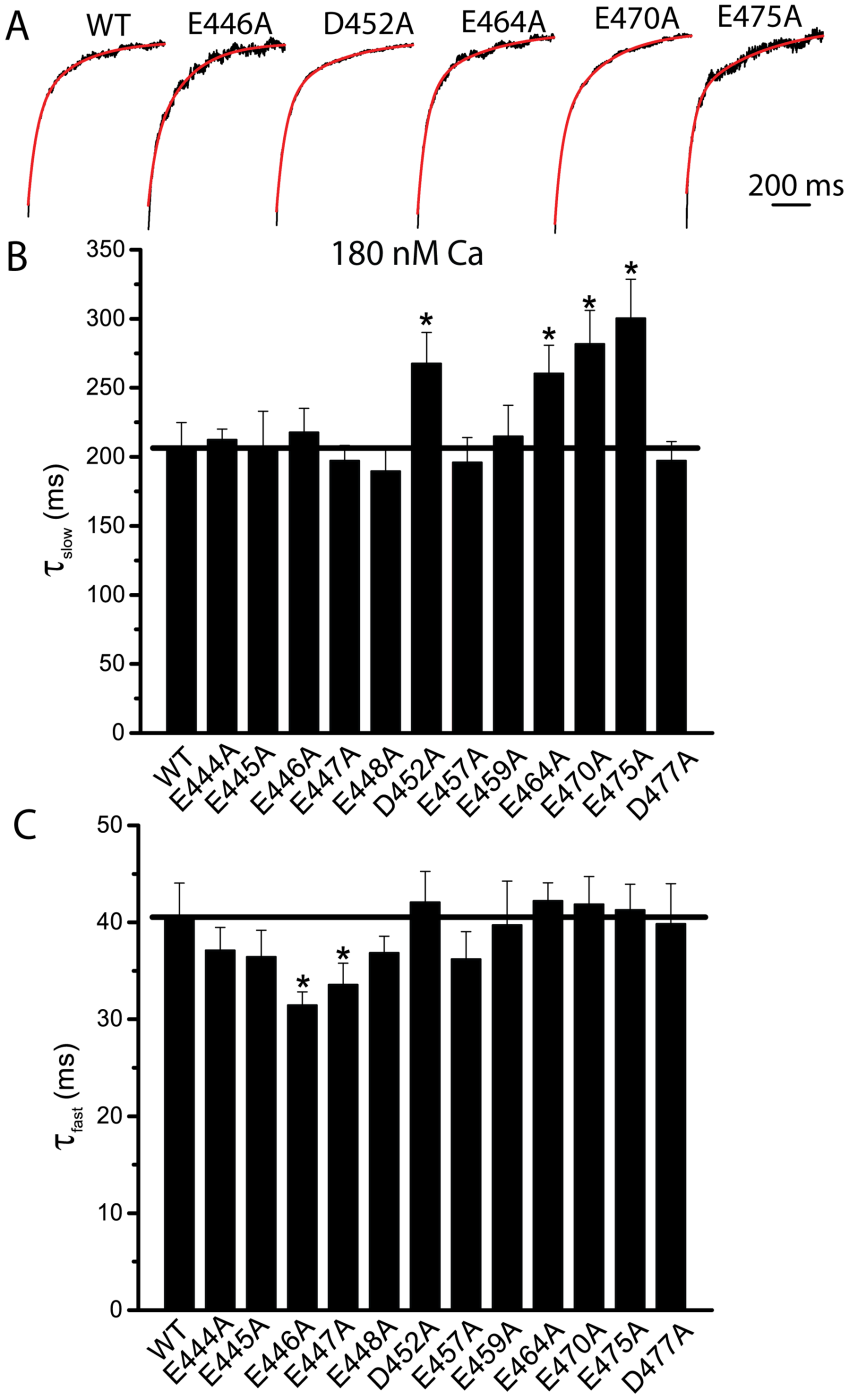
Activation kinetics of single alanine substituted mutations. A. Representative currents of WT, E446A, D452A, E464A, E470A, and E475A Ano1, activated by 180^2+^ at +100 mV from a holding potential of 0 mV. The currents were fitted to two exponentials (superimposed in red lines). B.C. The time constant of the slow (B) and fast (C) components of wild type and each single alanine substituted mutation activated by 180 nM Ca^2+^ at +100 mV from a holding potential of 0 mV. n = 4–8 cells.

To test which of the acidic amino acids in the first cellular loop affected voltage- and calcium-dependent gating of the channel, we further substituted each negatively charged amino acids in and near the consecutive glutamates with single alanine. We first tested the kinetics of current activation for each mutation. Compared with WT, D452A, E464A, E470A, and E475A mutations exhibited an increase in the τ_slow_ by 62, 35, 76, and 95 ms, respectively (Fig. 3A,B). The τ_slow_ of D452A, E464A, E470A, and E475A mutations was significantly slower than that of the WT, suggesting that these mutations may alter Ca^2+^-dependent gating of the channel. The τ_slow_ were changed by less than 30 ms for all the other mutations compared with WT, and did not significantly different from that of WT (Fig. 3A,B). E444A, E445A, E446A, E447A, E448A, and E457A mutations showed a decrease in the τ_fast_ (Fig. 3A,C). Only E446A and E447A mutations exhibited a significant decrease in the τ_fast_. These results suggested that E444A, E445A, E446A, E447A, E448A, and E457A mutations may alter voltage-dependent activation of the channel. The τ_slow_ and τ_fast_ of E459A and D477A mutations were similar to those of the WT, suggesting that E459A and D477A mutations did not alter Ca^2+^- and voltage-dependent gating of the channel.

**Figure 4:**
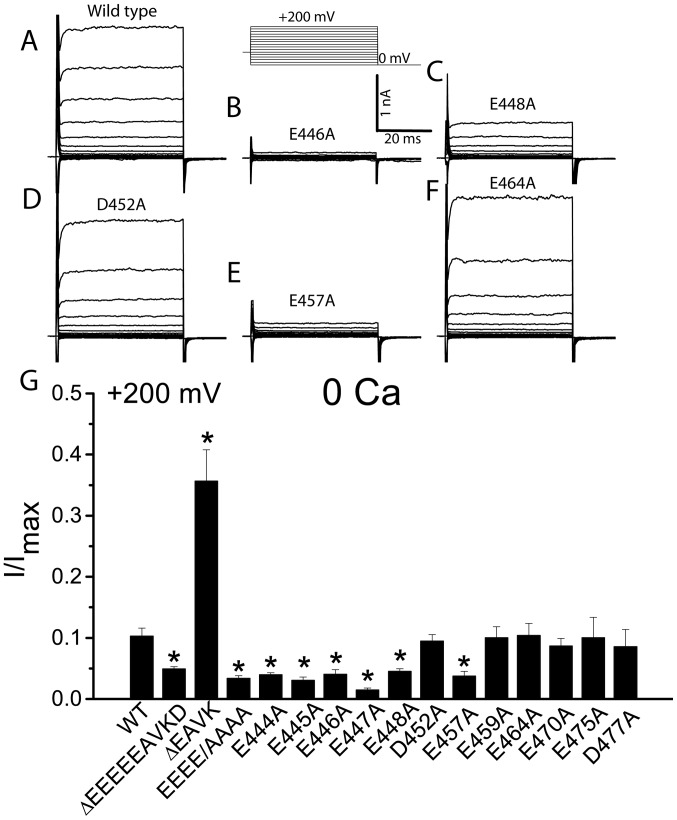
Voltage-dependent activation of Ano1. A–G. Representative traces of Ano1 activated by nominal “0” Ca^2+^ for wild type (A), E446A (B), E448A (C), D452A (D), E457A (E), and E464A (F). Cells were voltage clamped by stepping from a holding potential of 0 mV to various potentials between −100 mV to +200 mV in 20 mV increment for 50 ms, following by a step to −100 mV (voltage protocol is shown above B). G. Effects of single alanine-substituted mutations on currents activated by depolarization in the absence of Ca^2+^. The steady state outward currents at +200 mV were normalized to the maximal currents activated by 25 µM Ca^2+^. n = 4–11 cell; *p<0.05 vs wild type.

We further examined the voltage-dependent activation of each alanine substituted mutation in the absence of Ca^2+^. In nominal 0 Ca^2+^, E444A, E445A, E446A, E447A, E448A, and E457A mutations had significant smaller current amplitudes at +200 mV than wild type, suggesting that these amino acids are critical for voltage-dependent activation of the channel. In contrast, D452A, E459A, E464A, E470A, E475A, and D477A mutations had similar current amplitudes at +200 mV compared with wild type (Fig. 4), suggesting that these acidic amino acids are not important for voltage sensing.

**Figure 5:**
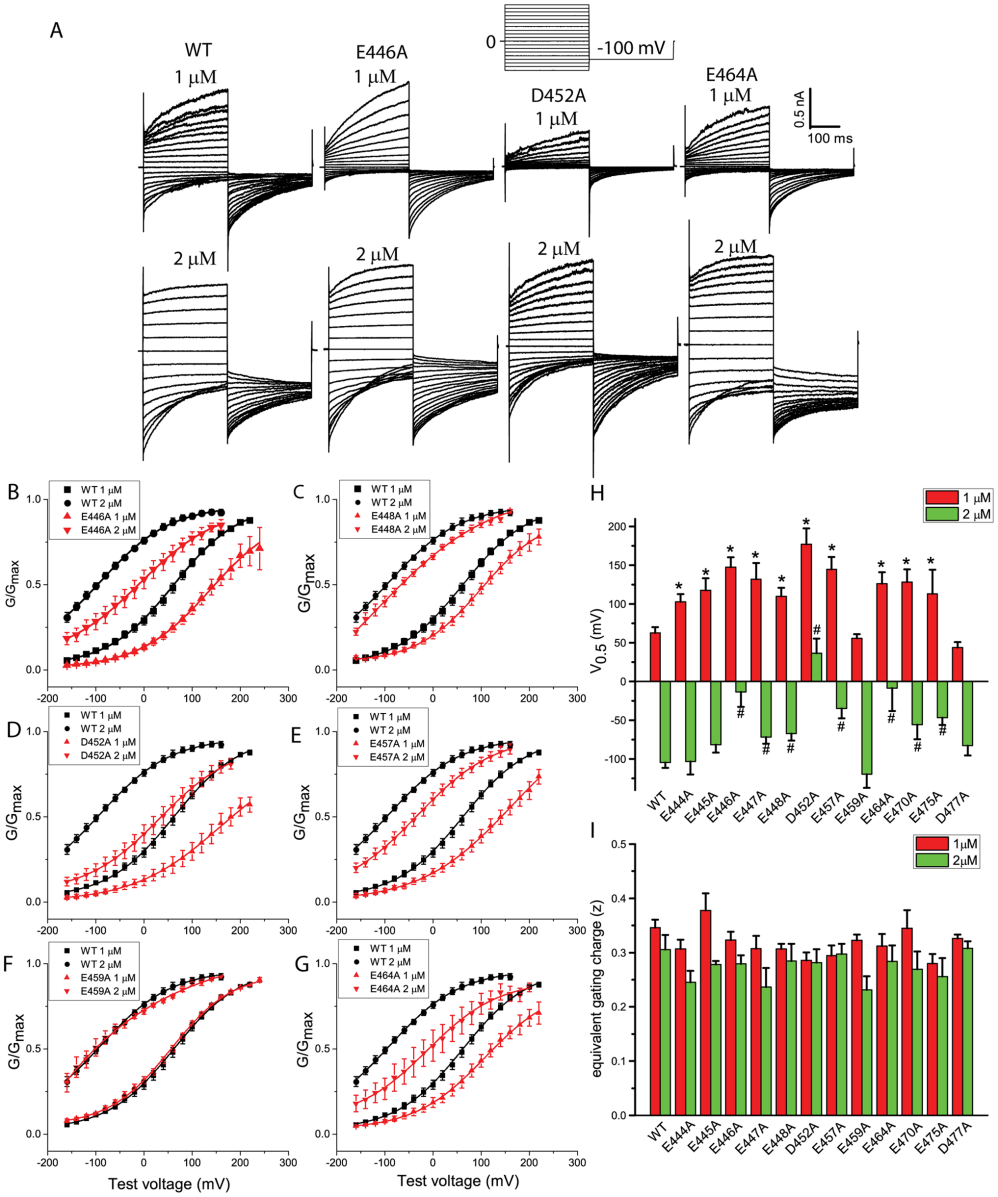
Activation of single alanine-substituted mutations by Ca^2+^ in excised patches. A. Representative currents of wild type, E446A, D452A, and E464A Ano1 activated by 1 µM and 2 µM Ca^2+^. Patches were voltage clamped with 300-ms voltage steps from −160 to +200 mV in 20-mV increments, following by a 300-ms step to −100 mV (voltage protocol shown above). B–G. Normalized G-V relations of wild type (black), as well as E446A (red, A), E448A (red, B), and D452A (red, C), E457A (red, D), E459A (red, E), and E464A (red, F) activated by 1 µM and 2 µM Ca^2+^. Each G-V curve was fitted to a Boltzmann function, and then normalized to the maximum of the fit. n = 5–11 cells. H.I. The V_0.5_ (H) and equivalent gating charge z (I) obtained from the G-V curve of each single alanine-substituted mutation. n = 5–11 cells; *p<0.05 vs wild type.

We further tested effects of [Ca^2+^] on the G-V relationships of Ano1 in excised patches. The WT G-V curve was fitted well by the Boltzmann equation with shallow voltage dependence. At 1 µM Ca^2+^, V_0.5_ = 63±7 mV (Fig. 5A,B,H). Doubling the [Ca^2+^] to 2 µM shifted the G-V curve to the left 167 mV (Fig. 5A,B,H). Single alanine mutation of the glutamates (E444A, E445A, E446A, E447A, E448A, and E457A) that exhibited decreases in voltage sensing (Fig. 4) showed a shift of V_0.5_ in the hyperpolarizing direction (Fig. 5A,B,C,E,H). However, these glutamates did not shift V_0.5_ equally. E446A showed the largest effects, while E444A had the smallest effects (Fig. 5H). For those acidic amino acids that exhibited no significant change in voltage sensing (Fig. 4), D452A, E464A, E470A, and E475A mutations, but not E459A and D477A mutations, showed a shift of V_0.5_ in the hyperpolarizing direction (Fig. 5A,D,F,G,H). These acidic amino acids may be responsible for sensing Ca^2+^.

We also examined the slope of the G-V curve by fitting with Boltzmann equation to evaluate the equivalent gating charge (z). For wild type, the z values at 1 µm Ca^2+^ and 2 µm Ca^2+^ were 0.35 and 0.31, respectively. Increased Ca^2+^ concentrations did not significantly altered z values. For all these mutations, we did not find a significant change in z values at either concentration (Fig. 5I).

**Figure 6:**
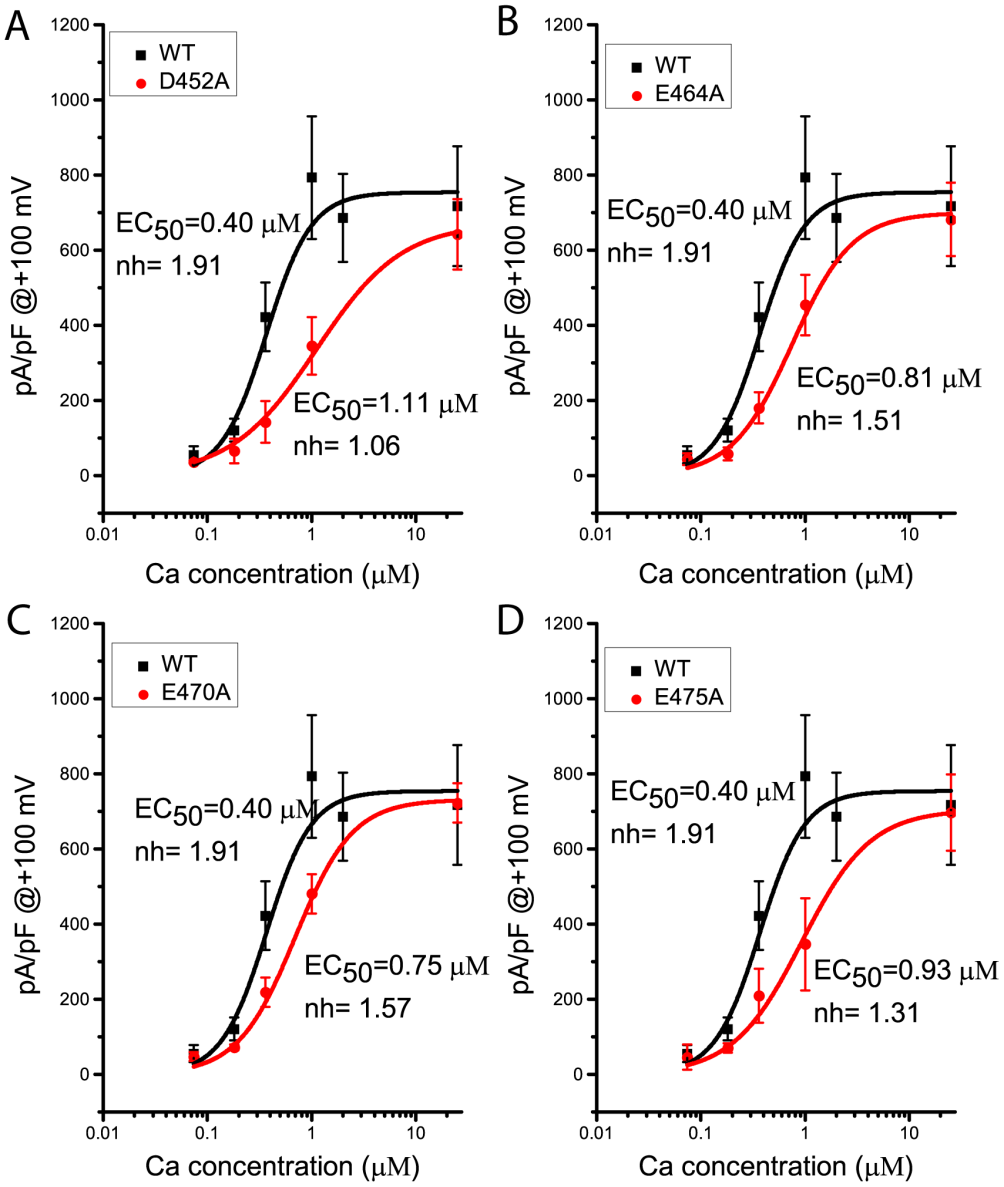
D452A, E464A, E470A, and E475A mutations decreased the calcium sensitivity. The steady state current densities at +100 were plotted versus Ca^2+^ concentrations from wild type (black), D452A (red, A), E464A (red, B), E470A (red, C), and E475A (red, D). The plots were fitted to Hill equations. n = 4–9 cells.

We further examined the calcium sensitivity of the D452A, E464A, E470A, and E475A mutations (Fig. 6 and S1). The apparent EC_50_ for Ca^2+^ was 1.11 µM for D452A, 0.81 µM for E464A, 0.75 µM for E470A, and 0.93 µM for E475A, which were approximately 2–3 fold higher than that of the WT (0.4 µM). These findings further confirmed that acidic residues D452, E464, E470, and E475 were important in Ca^2+^ -dependent gating of Ano1.

## Discussion

Ano1 has been identified as a novel CaCC that plays key roles in many physiological processes [2], [3], [29]. Ano1 exhibits voltage-and Ca^2+^-dependent activation [24], [25]. However, the structural determinant in Ano1 for voltage and calcium sensing remains unclear. The first intracellular loop is characterized by five consecutive glutamates (_444_EEEEE_448_) and _448_EAVK_451_, which is lacking in the splice variant c. We have previously found that the _444_EEEE/AAAA_447_ mutation inhibits the voltage-dependent activation of Ano1 without changes in the apparent Ca^2+^ sensitivity, whereas the ΔEAVK mutation decreased the apparent Ca^2+^ sensitivity with enhanced voltage-dependent activation of Ano1 [24], indicating that the first intracellular loop is critical for voltage- and Ca^2+^-dependent gating of Ano1. In agreement with our previous finding, the present study further showed that deletion of _444_EEEEEAVKD_452_ produced a more than 40-fold decrease in the apparent Ca^2+^ sensitivity, accompanied with slow kinetics of channel activation. By analyzing the kinetics of current activation at different Ca^2+^ concentrations for the WT and _444_EEEE/AAAA_447,_ ΔEAVK, and Δ_444_EEEEEAVKD_452_ mutations, we found that τ_fast_ and τ_slow_ of the channel were associated with voltage- and Ca^2+^-dependent activation of Ano1, respectively. Among the single alanine substituted mutations, E444A, E445A, E446A, E447A, E448A, and E457A mutations showed a decreased in the τ_fast_, suggesting that these glutamates are involved in voltage gating of the channel. In agreement with this idea, these mutations significantly inhibited voltage-dependent activation of Ano1 in the absence of Ca^2+^, and greatly shifted the G-V curve to the right. Because we have previously found that the _444_EEEE/AAAA_447_ mutation does not alter Ca^2+^ sensitivity, we believe that these glutamates (E444, E445, E446, E447, E448, and E457) are involved in voltage-gating of Ano1. Furthermore, we found that D452A, E464A, E470A, and E475A mutations exhibited an increase in the τ_slow_. Because these mutations did not alter voltage-dependent activation of the channel, but significantly decreased Ca^2+^ dependence of G-V curve and produced a 2–3 fold decrease in the calcium sensitivity, suggesting that they may be involved in Ca^2+^-gating of Ano1. Our findings demonstrate that the acidic amino acids in the first intracellular loop likely contribute to both voltage- and Ca^2+^-gating of Ano1.

### Voltage-dependent Gating of Ano1

Though we identify E444, E445, E446, E447, E448 and E457 as the critical residuals for voltage sensing, a lack of structural information precludes insight into how these residues are related to the voltage sensor. This region has been demonstrated to be cytoplasmic in both Ano1 and Ano7 [26], [30]. Thus, this region is highly unlikely to reside within the voltage field of the membrane and therefore is unlikely to directly sense voltage. In addition, if these acidic residues directly sense voltage, we would expect that each amino acid with the same negative charge should contribute equally to voltage sensing. However, the V_0.5_ shift in the G-V curve is different for each alanine substituted mutation, with V_0.5_ shifts most for E446A and least for E444A. Furthermore, mutations in the voltage sensor alter equivalent gating charge as measured as slope in G-V curve. However, all these mutations tested in this study do not alter the slope in G-V curve, further suggesting that they indirectly regulate a voltage sensor. The equivalent gating charge is about 0.33, about 4–6 fold less than that of voltage gated ion channel such as BK channel, suggesting that structure of the voltage sensor is different from that of voltage gated ion channels. Because the voltage sensitivity of Ano1 is very shallow, it seems unlikely that the voltage sensor is comprised of charged amino acids within a transmembrane segment like voltage-gated cation channels. The sequence of Ano1 provides no clear clues where the voltage sensor might be located. There are 3 basic amino acids at the interface between transmembrane domain 2 and the first intracellular loop, but mutation of these does not significantly affect Ca^2+^-independent gating (data not shown).

It remains unknown how the first intracellular loop may couple to channel gating, because the location of the channel gate and selectivity filter remains to be established. The pore of Ano1 has been proposed to be located in a re-entrant loop between transmembrane domains 5 and 6, because mutations in this region alter the relative anion/cation selectivity of the channel [31], [32]. Recently, a revised model of Ano1 suggests that that re-entry loop does not exist [26]. It is possible that other transmembrane domains such as transmembrane domain 2 may also contribute to channel permeation. The acidic amino acids in the first intracellular loop may contribute to stabilize the permeant anions occupancy of the pore, which has been shown to regulate voltage gating of the channel [24]. This hypothesis is supported by the finding that mutations with decreased voltage activation (such as E447A vs WT) causes a right shift in the G-V curve like anions with a low occupancy (Cl^−^ vs SCN^−^ or NO_3_^−^) in the pore [24]. If the hypothesis that regulation of channel permeation pathway changes voltage gating of the channel is true, we would expect that Ano1 with altered voltage-dependent activation should exhibit different anion permeability. In agreement with this hypothesis, Ferrera et al. reported that Ano1(0), a minimal isoform of Ano1 that completely lacked voltage-dependent activation, exhibited a large anion permeability than Ano1 (*abc*) [33].

Mutation or deletion of the cluster of five consecutive glutamates (_444_EEEEE_448_) significantly reduced voltage-dependent activation [24], and decreased τ_fast_ without significant change in τ_slow_ (Fig. 2). Single alanine-substitution of these glutamates and the E457A mutation produced a similar effect on τ_fast_, but to a less extent. These mutations may induce the channel to stabilize in the closed conformation, thus reducing voltage-dependent activation of the channel. This hypothesis seems to be supported by the findings that these mutations without altered Ca^2+^ sensitivity shifted the G-V curve to the right. Stabilization in the closed confirmation may affect the permeant anion occupancy of the pore, which has been reported to regulate voltage-dependent gating of heterologously expressed Ano1 and native CaCCs [24], [34], [35]. If this is true, it is reasonable to hypothesize that other domains of Ano1 that contribute to regulate channel conformational changes may modulate voltage-dependent gating of Ano1. This idea appears to be supported by the report that chimeras in which the transmembrane domain 7–8 or C-terminus of Ano1 was replaced with the equivalent domains of Ano2 exhibited faster activation kinetics compared with Ano1 [36].

The cluster of five consecutive glutamates (_444_EEEEE_448_) is also present in Ano2. Similar to Ano1, deletion of the five glutamates in Ano2 resulted in a right shift of the G-V curve, and modified voltage-dependent gating of the channel [37]. Due to the similar function of the first intracellular loop between Ano1 and Ano2, it is expected to find that a chimera in which the first intracellular loop of Ano1 was replaced with the equivalent domains of Ano2 did not alter channel properties [36]. In addition, Ano2 exhibited faster activation kinetics compared with Ano1 [36]. The C-terminus or transmembrane domain 7–8 may be responsible for faster activation kinetics in Ano2 [36].

### Ca^2+^-dependent Gating of Ano1

We have previously found that the splice variant c of Ano1 (ΔEAVK) exhibits an approximately 50-fold decrease in the Ca^2+^-sensitivity [24]. In the present study, we showed that the Δ_444_EEEEEAVKD_452_ mutation produced a similar decrease in the Ca^2+^ sensitivity, further suggesting that deletion of EAVK, not its adjacent acidic amino acids, contributes to the major effect of decreased Ca^2+^ sensitivity. This finding agrees with our previous report that the _444_EEEE/AAAA_447_ mutation did not alter Ca^2+^ sensitivity [24]. Because the E448A mutation did not alter the Ca^2+^ sensitivity greatly, the backbone carbonyl groups of the EAVK are likely to be the key Ca^2+^-coordinating oxygen atoms in the Ca^2+^ binding site. It has been reported that deletion of VK, not EA, generates currents similar to deletion of all four residues EAVK [25], suggesting that the backbone carbonyl oxygen atoms of VK may be involved in Ca^2+^ coordination. In addition, we found that D452A, E464A, E470A, and E475A mutations, which did not alter voltage-dependent activation, exhibited an increase in the Ca^2+^-dependent τ_slow_, significantly decreased Ca^2+^-dependence of the G-V curve, and produced a 2–3 fold decrease in the calcium sensitivity, suggesting that D452, E464, E470, and E475 are likely to be involved in Ca^2+^-coordination. However, the effects of these single alanine substituted mutations produced much less effects of [Ca^2+^] on the shift in the G-V curve compared with ΔEAVK: for ΔEAVK, 2 µM Ca^2+^ produced a similar shift in the G-V curve to 1 µM Ca^2+^ [24], whereas for these single alanine substituted mutations, 2 µM Ca^2+^ shifted the G-V curve to the left by more than 140 mV compared with 1 µM Ca^2+^ (Fig. 5). These results suggest that the side chain carboxyl oxygen of these acidic residues may not be the key Ca^2+^-coordinating oxygen atoms. However, we can not exclude the possibility that these acidic residues D452, E464, E470, and E475, are not involved in direct Ca^2+^ coordination, but play a role in modulating the Ca^2+^ affinity in their adjacent Ca^2+^ -binding site.

Recently, in a revise model of Ano1, the two acidic residues E702 and E705 in the third intracellular loop have been identified to be critical for the Ca^2+^ sensitivity of Ano1 [26]. Mutations in the two acidic residues caused approximately 100-fold decreases in the Ca^2+^ sensitivity [26]. The importance of the acidic residues in the Ca^2+^ sensitivity has been confirmed in Ano2 and Ano6 [36], [38]. Because Ca^2+^ is commonly coordinated by 6–8 oxygen atoms [39], other acidic residues are likely to contribute to the binding site. Taken together with the present study, it is possible that the backbone carbonyl oxygen atoms of the EAVK and the side chain carboxylic oxygen atoms of the acidic residues D452, E464, E470, and E475 in the first intracellular loop, and E702 and E705 in the third intracellular loop may coordinate Ca^2+^ in the Ca^2+^ binding site in Ano1. However, we can not exclude the possibility that the acidic residues in the first and third intracellular loops contribute to separate Ca^2+^ binding sites. Although it is possible that Ano1 has more than one type of Ca^2+^ binding site, there is no direct evidence to support this.

Recent studies have shown that calmodulin (CaM) can directly bind and regulate Ano1 [22], [23]. Tian et al. have reported that a CaM-binding site that overlaps with the b splice segment is required for Ano1 (*abc*) channel activation by Ca^2+^ [22]. However, Yu et al. reported that CaM did not immunoprecipitate with Ano1(*abc*), and activation of Ano1 by Ca^2+^ is not mediated by CaM [40]. It remains controversial whether the b segment in Ano1(*abc*) mediates channel activation by Ca^2+^/CaM. Jung et al. found that CaM modulated HCO_3_^−^ permeability of Ano1(*ac*) that did not contain the b segment, but did not alter the ability of Ca^2+^ to activate the channel [23]. Furthermore, Yu et al. found that CaM did not mediate activation of Ano1(*ac*) by Ca^2+^ [40]. It seems that activation of Ano1(*ac*) by Ca^2+^ is not mediated by CaM, but via the calcium binding sites on the channel. In the present study, our findings that mutations in acidic residues D452, E464, E470, and E475 altered the apparent calcium sensitivity of Ano1(*ac*) further suggest that these acidic residues may contribute to Ca^2+^-binding site on the channel. Since Ano1(*ac*) can interact with many Ca^2+^-binding proteins [41], it is possible that accessary proteins also play a role in Ca^2+^ -dependent gating of Ano1.

Ano2 has been identified as a CaCC in the photoreceptors [42], and olfactory sensory neurons [14], [43], and hippocampal neurons [44]. The apparent affinity for Ca^2+^ of Ano2 is lower compared with Ano1 [36], [37]. Since only deletion of the c segment (EAVK) reduced the Ca^2+^ sensitivity greatly, it is possible that the backbone carbonyl groups of the EAVK contribute to the Ca^2+^-coordinating oxygen atoms in the Ca^2+^ binding site. If this is true, it is reasonable to hypothesize that the difference in the sequence of the corresponding residues (ERSQ) in Ano2 may not contribute to the difference in the Ca^2+^ sensitivity between Ano1 and Ano2. Consistent with our idea, Scudieri et al. found that replacement of the first intracellular loop of Ano1 with the corresponding domains of Ano2 did not alter the apparent Ca^2+^ sensitivity [36]. Other domains such as the third intracellular loop may be responsible for different Ca^2+^ sensitivity between Ano1 and Ano2 [36]. In addition, it has been reported that the alternative N terminus alters the apparent Ca^2+^ sensitivity of Ano2 [43]. It appears that multiple domains may contribute to the different Ca^2+^ sensitivity between Ano1 and Ano2.

In summary, the present study supports the conclusion that the first intracellular loop is critical for voltage- and calcium-dependent gating of Ano1. The backbone carbonyl oxygen atoms of the EAVK are likely to play a key role in Ca^2+^ coordination. Among the acidic residues adjacent to EAVK, the cluster of five consecutive glutamates (_444_EEEEE_448_) and E457 are important for voltage-dependent gating of the channel. Other acidic residues D452, E464, E470, and E475 are involved in Ca^2+^ -dependent gating of Ano1. The present study identifies that acidic residues in the first intracellular loop is the important structural determinant that couples the voltage- and calcium-dependent gating of Ano1.

## Supporting Information

Figure S1 **Representative traces of D452A (A), E464A (B), E470A(C) and E475A (D) activated by Ca^2+^ concentrations ranging from 74 nM to 25 µM.** Cells were voltage clamped from a holding potential of 0 mV to various potentials between −100 mV to +100 mV in 20 mV increments for 700 ms, followed by a 100-ms step to −100 mV.
(TIF)
