# Correction: Acidic Amino Acids in the First Intracellular Loop Contribute to Voltage- and Calcium- Dependent Gating of Anoctamin1/TMEM16A

**DOI:** 10.1371/journal.pone.0107343

**Published:** 2014-09-02

**Authors:** The PLOS ONE Staff

The following information is missing from the Funding section: QX and YC are supported by NIH grants GM60448 and EY014852 to Criss Hartzell at the Department of Cell Biology, Emory University.

## References

[1] Xiao Q, Cui Y (2014) Acidic Amino Acids in the First Intracellular Loop Contribute to Voltage- and Calcium- Dependent Gating of Anoctamin1/TMEM16A. PLoS ONE 9(6): e99376 doi:10.1371/journal.pone.0099376 24901998 10.1371/journal.pone.0099376 PMC4047086

