# Supplementary figures and images for: Acidic Amino Acids in the First Intracellular Loop Contribute to Voltage- and Calcium- Dependent Gating of Anoctamin1/TMEM16A
Source: PLoS One. 2014 Jun 5;9(6):e99376. doi: 10.1371/journal.pone.0099376 (PMC4047086; doi:10.1371/journal.pone.0099376)

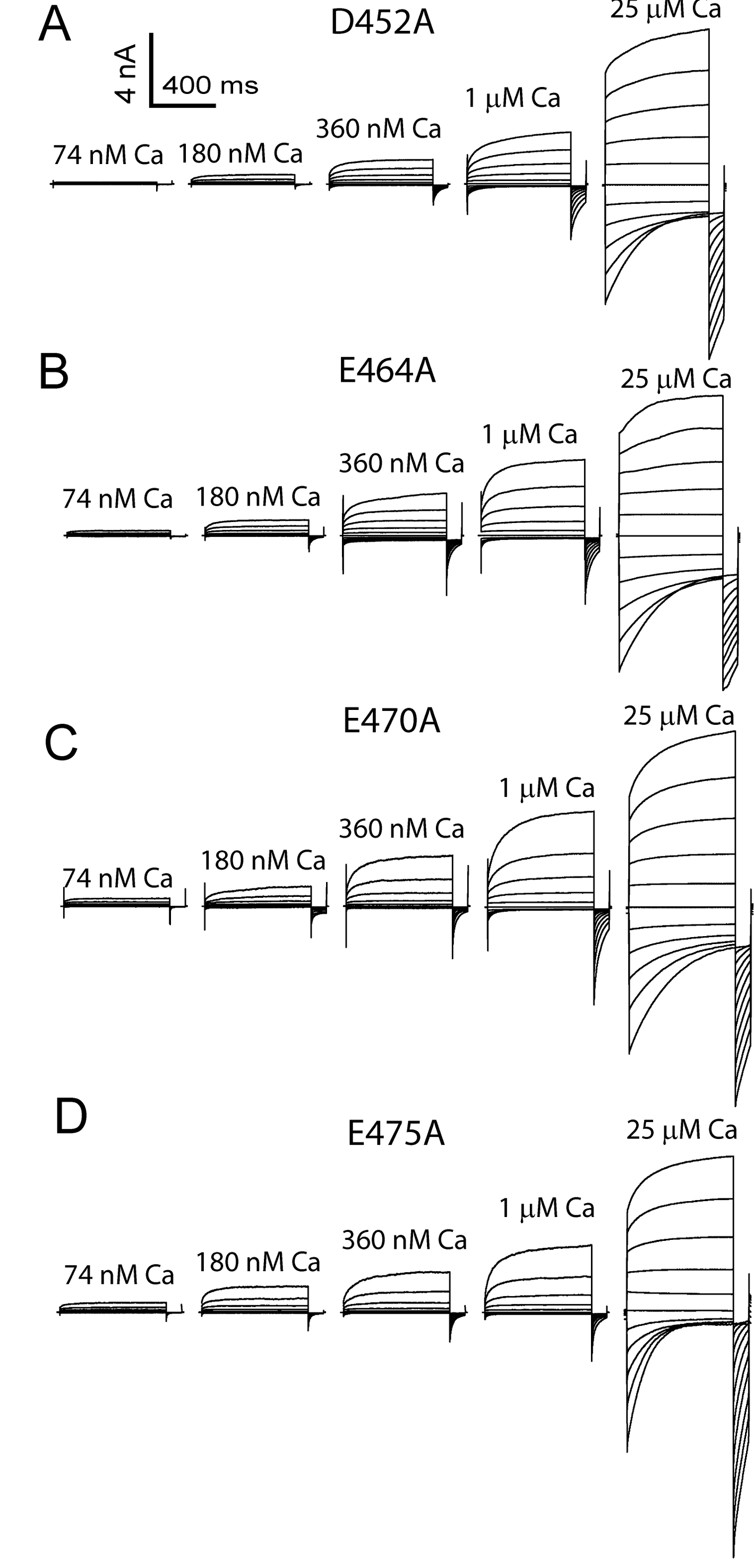

Supplement: Figure S1 — Representative traces of D452A (A), E464A (B), E470A(C) and E475A (D) activated by Ca2+ concentrations ranging from 74 nM to 25 µM. Cells were voltage clamped from a holding potential of 0 mV to various potentials between −100 mV to +100 mV in 20 mV increments for 700 ms, followed by a 100-ms step to −100 mV. (TIF) [file pone.0099376.s001.tif]
